# Travel patterns and demographic characteristics of malaria cases in Swaziland, 2010–2014

**DOI:** 10.1186/s12936-017-2004-8

**Published:** 2017-09-08

**Authors:** Natalia Tejedor‐Garavito, Nomcebo Dlamini, Deepa Pindolia, Adam Soble, Nick W. Ruktanonchai, Victor Alegana, Arnaud Le Menach, Nyasatu Ntshalintshali, Bongani Dlamini, David L. Smith, Andrew J. Tatem, Simon Kunene

**Affiliations:** 1 WorldPop, University of Southampton, Southampton, UK; 2 National Malaria Control Programme, Manzini, Swaziland; 3 Flowminder Foundation, Stockholm, Sweden; 4 Clinton Health Access Initiative, Boston, MA USA; 5 Institute for Health Metrics and Evaluation, University of Washington, Seattle, USA

**Keywords:** Imported malaria, Travel history, Malaria elimination, Reactive case detection, Surveillance system

## Abstract

**Background:**

As Swaziland progresses towards national malaria elimination, the importation of parasites into receptive areas becomes increasingly important. Imported infections have the potential to instigate local transmission and sustain local parasite reservoirs.

**Methods:**

Travel histories from Swaziland’s routine surveillance data from January 2010 to June 2014 were extracted and analysed. The travel patterns and demographics of rapid diagnostic test (RDT)-confirmed positive cases identified through passive and reactive case detection (RACD) were analysed and compared to those found to be negative through RACD.

**Results:**

Of 1517 confirmed cases identified through passive surveillance, 67% reported travel history. A large proportion of positive cases reported domestic or international travel history (65%) compared to negative cases (10%). The primary risk factor for malaria infection in Swaziland was shown to be travel, more specifically international travel to Mozambique by 25- to 44-year old males, who spent on average 28 nights away. Maputo City, Inhambane and Gaza districts were the most likely travel destinations in Mozambique, and 96% of RDT-positive international travellers were either Swazi (52%) or Mozambican (44%) nationals, with Swazis being more likely to test negative. All international travellers were unlikely to have a bed net at home or use protection of any type while travelling. Additionally, paths of transmission, important border crossings and means of transport were identified.

**Conclusion:**

Results from this analysis can be used to direct national and well as cross-border targeting of interventions, over space, time and by sub-population. The results also highlight that collaboration between neighbouring countries is needed to tackle the importation of malaria at the regional level.

**Electronic supplementary material:**

The online version of this article (doi:10.1186/s12936-017-2004-8) contains supplementary material, which is available to authorized users.

## Background

Global malaria eradication is back on the international agenda [1, 2], with many countries making malaria elimination a national goal [3]. The past decade has seen significant declines in malaria prevalence [4, 5], with the risk of acquiring malaria reduced by 37% since 2000 and the risk of dying from the disease decreasing by 60% [6].

Achieving elimination requires a re-orientation from the universal prevention and treatment measures that typically define a control or surveillance programme towards targeted operations, such as identifying residual transmission foci, identifying and curing both asymptomatic and symptomatic infections, focusing vector control or parasite-based attack measures to high-risk areas and managing importation risk. Understanding human movement, which can provide connections between disparate high transmission or receptive areas [7], is important for designing appropriate elimination strategies and avoiding resurgence in post-elimination settings [8, 9]. Additionally, the identification of the sources of imported infections play a crucial role in malaria programme pre-elimination and elimination phases, through the assessment of patient travel history records [10], which makes possible the identification of imported cases and rates, importation routes and the delimitation of areas with high-risk travellers and sources [8, 9, 11].

Swaziland is a small, landlocked country in southern Africa, and due to elevation and climatic conditions the majority of Swaziland has historically had low levels of malaria transmission [12]. The scale-up of interventions and the expansion of surveillance in recent years, incorporating active, reactive and proactive case detection, have brought the country close to elimination [12–15]. Between 2000 and 2014 Swaziland reported a decrease of more than 97% in malaria cases and a reduction of over 93% in malaria deaths between 2001 and 2014 [6]. However, population movements, both within and outside the country, especially to malaria-endemic areas, are a hindrance to elimination success [16, 17]. Understanding the levels of malaria importation, the sources and routes taken by infected travellers, and the characteristics of infected travellers compared to those who are uninfected is valuable for guiding elimination strategies and designing interventions to achieve and sustain malaria elimination [7, 18].

This article describes the characteristics of all individuals passively detected by health facilities and all those surveyed in follow-on reactive case detection (RACD) carried out between January 2010 and June 2014 and map the reported domestic and international travel histories. It also explores the differences in travel patterns between confirmed positive cases, with those found to be negative, and examined demographic, socio-economic and geographic factors associated with any differences.

## Methods

**Fig. 1:**
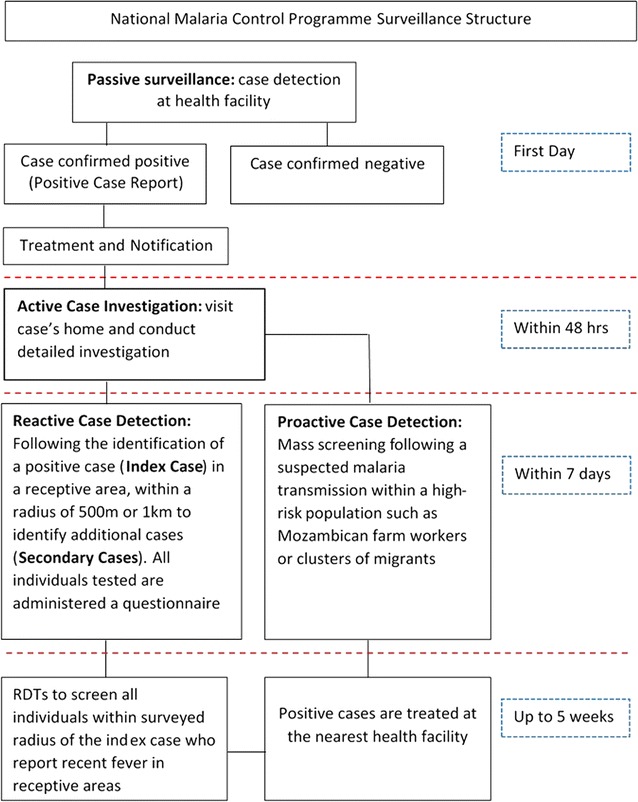
Swaziland National Malaria Control Programme Surveillance structure (Adapted from the NMCP surveillance manual [19])

In 2009, the Swaziland National Malaria Control Programme (NMCP) introduced active surveillance, consisting of: (1) active case investigation at the household of the index case, triggered by parasitological confirmation of a malaria case at a health facility; (2) RACD, triggered by the location of a confirmed malaria case in Swaziland’s receptive area; and, (3) pro-active case detection, triggered by strong suspicion of malaria transmission within a defined detection area and on high-risk populations, such as Mozambican farm workers [19] (Fig. 1). Every case that is parasitologically confirmed at health facilities (‘index case’), whether it is identified by rapid diagnostic test (RDT) and/or by microscopy, is investigated at the patient’s home within 7 days (January 2010 to June 2013) or 48 h (July 2013 to June 2014) of the patient’s presentation date, subject to consent by the patient or guardian. If the confirmed malaria case lives in Swaziland’s receptive area (approximately the eastern half of the country), every person residing within a radius of either 1 km (January 2010 to June 2013) or 500 m (July 2013 to June 2014) from the residence of the index case is tested for malaria. A RACD event remains open for up to 5 weeks, where the NMCP additionally conducts fever screening and where individuals near the index case report a recent fever, enabling identification of any additional malaria cases (‘secondary cases’). Any identified positive case is referred (often driven) to the nearest health facility for treatment and followed up [19]. Figure 1 outlines the surveillance structure.

During both index case investigation and RACD, the surveillance agent is required to question each participant on their age, gender, occupation, and nationality. Additionally, travel history is recorded, including places visited during the 8 weeks prior to the investigation, duration of stay, reason for travel, means of transport, border crossing sites, and access to/utilization of vector control and personal protection measures. The latitude and longitude of the residence of each individual is also recorded. Once the case investigation is undertaken, a case review is carried out to identify the origin of the infection and classify cases into the following groups: (1) imported: a case for which the origin can be traced to a known malarious area outside the country in which the case was diagnosed; (2) local: a case for which the origin is likely from local transmission; (3) intraported: a case which has its origins in another part of Swaziland; and, (4) unknown: where the information available does not enable a case classification [19].

The data used in this study are from the Swaziland Malaria Surveillance Database System (MSDS) and include data recorded between January 2010 and June 2014 for all index cases and between August 2012 and June 2014 for all individuals tested for malaria as part of RACD in pre-defined receptive areas. The data covers four full malaria seasons defined by the Swaziland NMCP: July 2010–June 2011, July 2011–June 2012, July 2012–June 2013, and July 2013–June 2014, with the addition of half a season: January 2010–June 2010. The data were anonymized and the place of residence of each individual and travel destination were georeferenced using ArcGIS v10.2.2 [20]. The geolocation of residences was aggregated by localities, which is the relevant operational unit at which public health decisions are made in Swaziland. The travel destinations outside Swaziland were identified to include city/town and country. For those locations in South Africa and Mozambique where the majority of international travel took place, the district level (administrative level 1) was also included. The international administrative units were obtained from the Global Administrative Areas Database (GADM) [21].

The surveillance data were cleaned by removing any potential duplicate or miscoded records, and any reactive case entries without a corresponding index case were excluded from the analyses. The data were then analysed using R [22], where descriptive statistics were used to create sociodemographic and travel profiles for each index case, and both the positive and negative cases identified though reactive case investigation. Logistic univariate and multivariate regressions were carried out to identify the relative risks factors associated to the travel patterns among all positive (index and secondary cases) and negative cases identified through RACD, not only in terms of sociodemographic characteristics but also protection used during travel and at home. The data from all index cases and individuals tested in RACD (positive and negative malaria confirmation) were split into three sets for further analyses in understanding travel patterns: (1) all those who travelled abroad; (2) those who travelled within Swaziland; and, (3) those who did not travel. The *Plasmodium falciparum* malaria prevalence map produced by the Malaria Atlas Project [5] was used to provide estimates of malaria transmission intensity at the travel destinations outside of Swaziland. Population distribution data were extracted from the WorldPop project [23] to quantify population densities at origin and destination locations. These datasets were in raster format, with a resolution of 0.0083 decimal degrees (approximately 1 km at the Equator) for 2010. Additionally, urban and rural extents were used to identify if the origin and/or travel destinations were urban and to estimate the distance to the nearest urban area, using a global urban map [24].

## Results

**Fig. 2:**
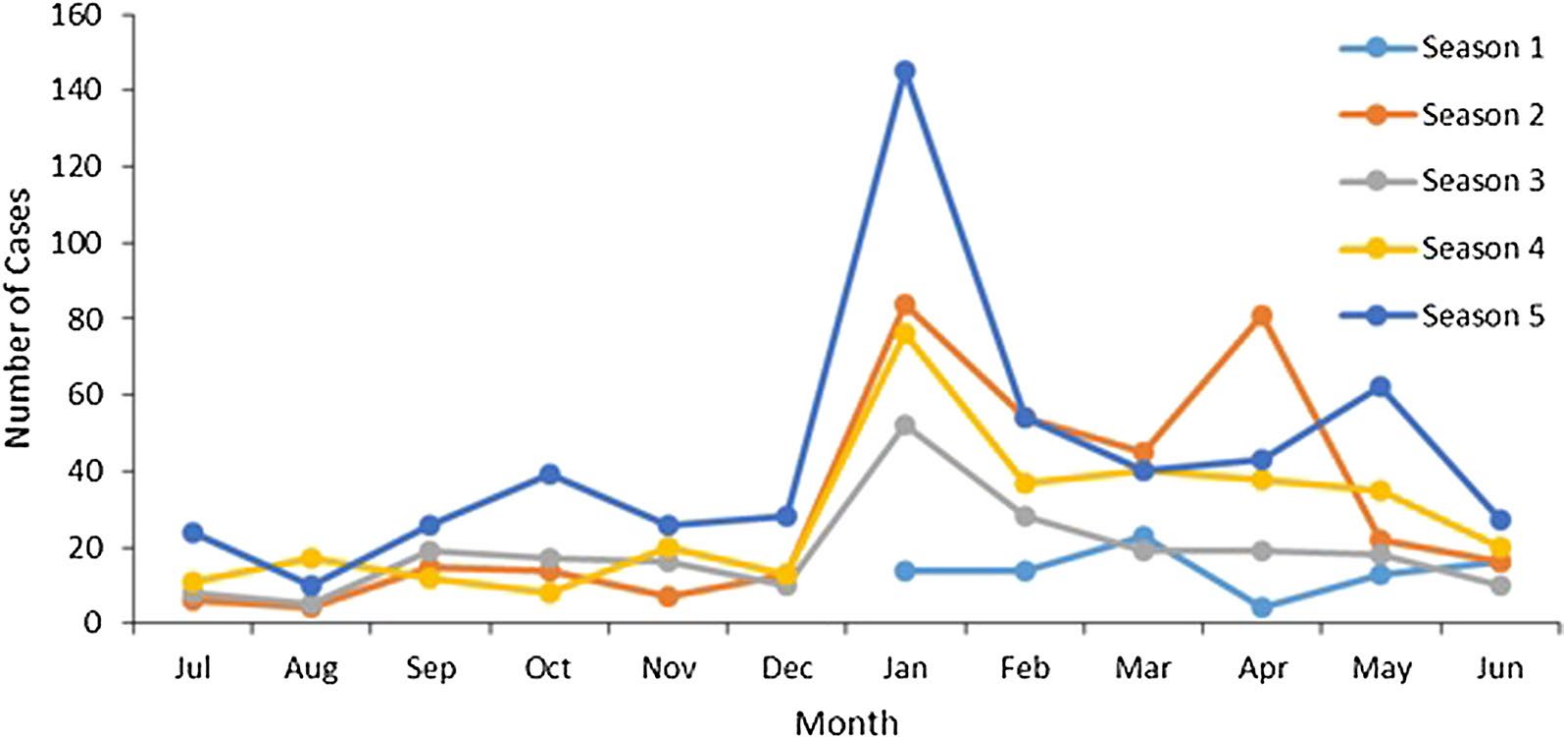
Number of positive cases (index cases) detected at health facilities per month by malaria season. *Season 1* Jan 2010–June 2010, *Season 2* July 2010–June 2011, *Season 3* July 2011–June 2012, *Season 4* July 2012–June 2013, *Season 5* July 2013–June 2014 (n = 1517)

The total number of confirmed malaria cases passively detected at health facilities between January 2010 and June 2014 was 2123. Active case investigation was successfully carried out on 1517 (71.5%) of these malaria positive cases (index cases). January had the largest number of index cases registered at the health facilities, with 371 (24.5%) cases, and August had the lowest number (36(2.3%)). Some 60% of cases were reported between January and April, and January 2014 had the largest number of cases reported overall, with 150 cases. Figure 2 depicts the number of cases by season and month.

In RACD events carried out between August 2012 and June 2014, a total of 9859 people were tested, leading to the identification of 105 new positive malaria cases via RDTs (secondary cases). Including both index and secondary cases, overall there were 1622 positive cases and 9754 negative cases. Positive cases identified through RACD were linked to 64 index cases. Forty-one (64%) of these index cases led to the identification of one secondary case, 15 (23%) to two secondary cases and five (8%) to three cases. Of the remainder, there were three (5%) index cases that led to the identification of five, six and eight additional cases, respectively. Some 44% of positive reactively detected cases were family members of index cases living in the same property or in the vicinity, and the remaining 56% of positive cases were detected in the households of neighbours within the surveyed radius of the index case.

Comparing the demographics of all negative and confirmed positive cases from RACD events, the latter were predominantly male (67%), while the former were mostly female (55%). Overall, the 24- to 44.9-year old age group had the highest number of cases, including both positive and negative cases (33% for positive cases and 25% for negative cases). Although the occupation for a large proportion of positive cases (42%) was not determined, the most commonly reported occupation for both positive and negative cases was ‘unemployed’ (16 and 34%, respectively), followed by ‘other’ (12 and 29%, respectively) and ‘student’ (12 and 26%, respectively). Furthermore, a large proportion of positive cases reported domestic or international travel history (65%) compared to negative cases (10%), (further information can be found in Additional file 1: Table S1). A total of 15 nationalities were reported among the positive cases, with the top nationalities being Swazi nationals (72%), Mozambican (26%) and South African (0.6%), while most of the negative cases were Swazi nationals (99%) with seven other nationalities being present.

**Fig. 3:**
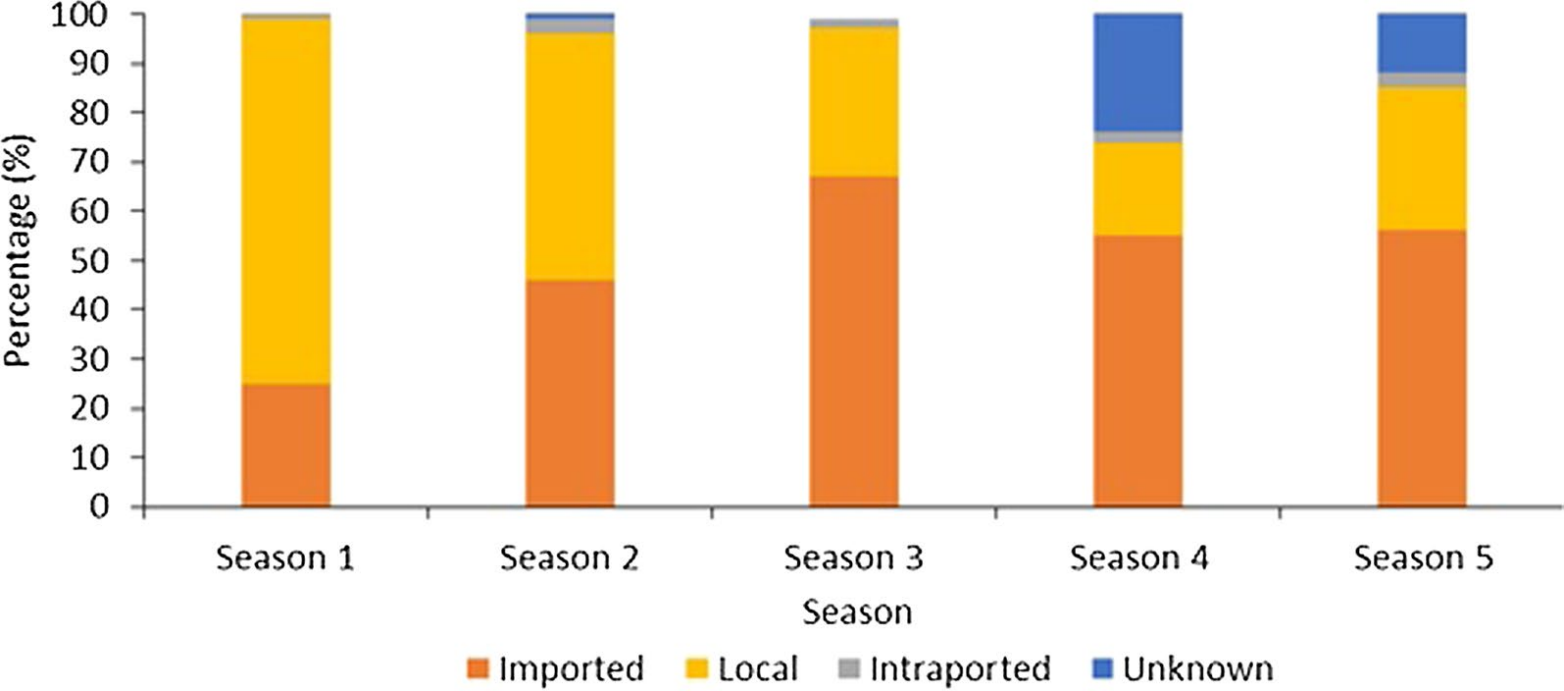
Classification of all positive cases [index cases (n = 1517) and secondary cases (n = 105)] the origin of malaria infection by season from January 2010 to June 2014. *Season 1* Jan 2010–June 2010 (n = 84), *Season 2* July 2010–June 2011 (n = 361), *Season 3* July 2011–June 2012 (n = 221), *Season 4* July 2012–June 2013 (n = 387), *Season 5* July 2013–June 2014 (n = 575)

Figure 3 shows the classification of the origin of malaria infection for all positive cases (index and secondary) by season. In total, there were 866 (53%) cases reported as imported, 548 (34%) as local, 42(3%) as intraported (from different areas within the country) and 166 (10%) as unknown. The first season of this analysis only had data from January to June 2010, and it showed the lowest percentage of cases classified as imported with 25% (21/84). From this period, the number of imported cases increased with July 2013 to June 2014 having the largest percentage of 56% (320/575). In contrast, the number of cases reported as local declined from 74% (62/84) of cases in between January 2010 to June 2010 to 29% (166/575) between July 2013 and June 2014.

### All travel

A total of 2056 people reported domestic or international travel history where individuals spent at least one night away. The malaria prevalence of those that travelled was 51%. A total of 2180 trips were reported, 1959 individuals made a single trip and the other 97 made at least one other trip (i.e., left home, returned and left again) or spent a night in an additional location while away (i.e., left home went to one destination, moved to another destination and returned). Thirty-one people (malaria prevalence of 90%) made multiple trips to destinations in both Swaziland and outside the country, some of which were within the same trip and some left home, returned and left again. The most common regions of residence among those travellers who tested positive were Manzini (43%) and Lubombo (34%).

Considering the risk factors for the outcome of malaria infection from travel, the univariate logistic regression indicated that travel is an important risk factor, especially travelling to multiple destinations within and outside the country (odds ratio (OR) 143.01 with 95% confidence intervals (CI) 50.5–599.3, p < 0.001). Travel outside the country increased the OR by 45.55 (CI 39.0–53.4, p < 0.001), and travel within Swaziland increased the OR by 3.22 (CI 2.6–3.91, p < 0.001). The risk factors that remained highly relevant in the multivariate analysis was being male and having travelled. Further details of the overall patterns can be found in Additional file 1: Tables S1 and S2. Additional details for those who did not travel can be found in Additional file 1: Table S3. The following sections describe the patterns of travel and risk factors of acquiring malaria with travel in more detail.

### International travel

**Fig. 4:**
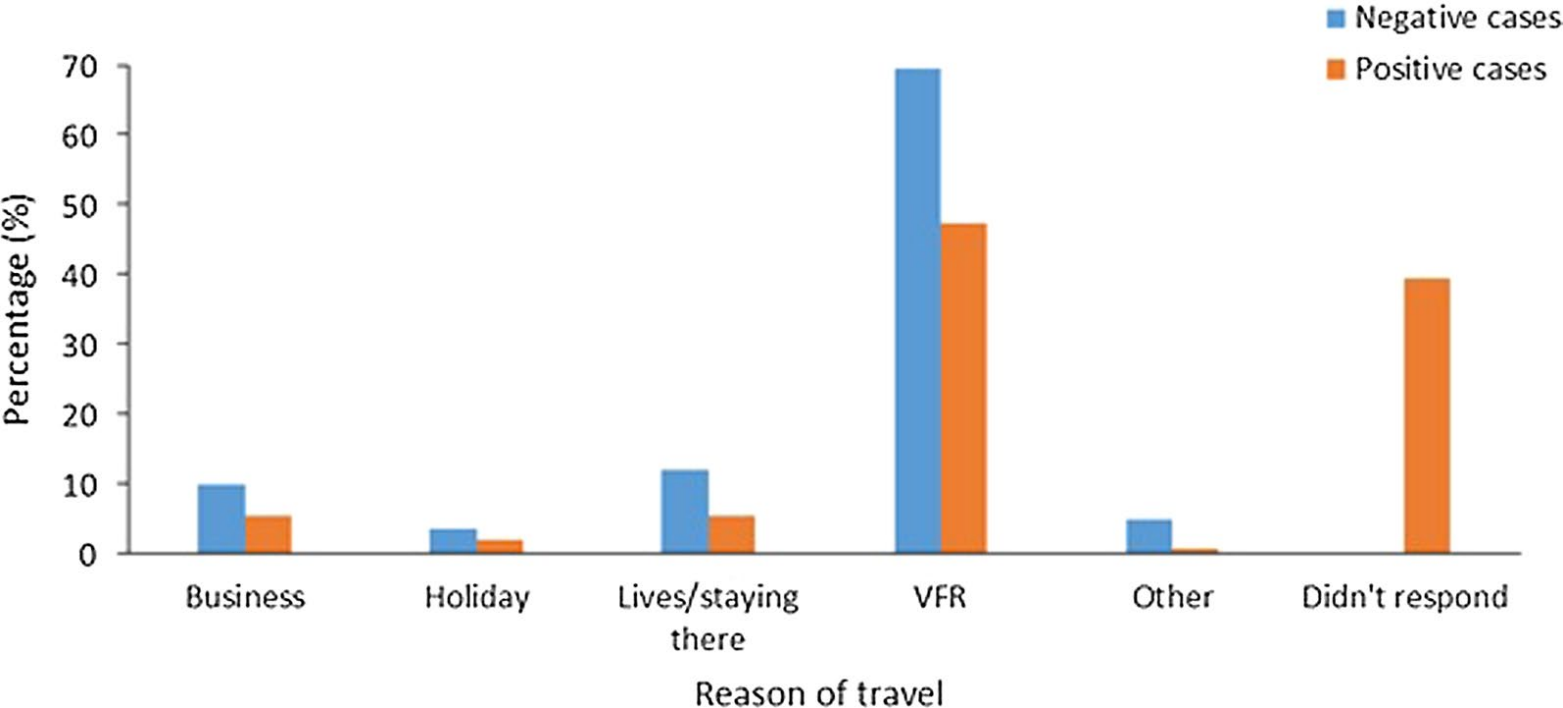
Reason for travel for all the trips made outside Swaziland. (*VFR* visiting friends and relatives) by negative (n = 306) and positive (n = 947) cases. Further information on the classifications can be found in Additional file 1: Table S4

A total of 1199 people travelled outside of Swaziland (malaria prevalence of 75%), making 1253 trips in total; 31 of these people also travelled within Swaziland at least once. The length of travel for 878 (70%) of these trips was specified, 6% spending one night away, 45% spending from two to ten nights away, 29% spent between 11 and 30 nights away, 11% between 31 and 100 nights, and 9% spending over 100 nights away. The most common reason for travel outside the country for both positive and negative cases was visiting friends and/or relatives with 58.2% of the total. A large percentage of positive cases (29%) did not specify the reason of travel. Figure 4 summarizes these data by reason for travel. The most common regions of residence among those travellers who tested positive were Manzini (46%) and Lubombo (31%).

**Fig. 5:**
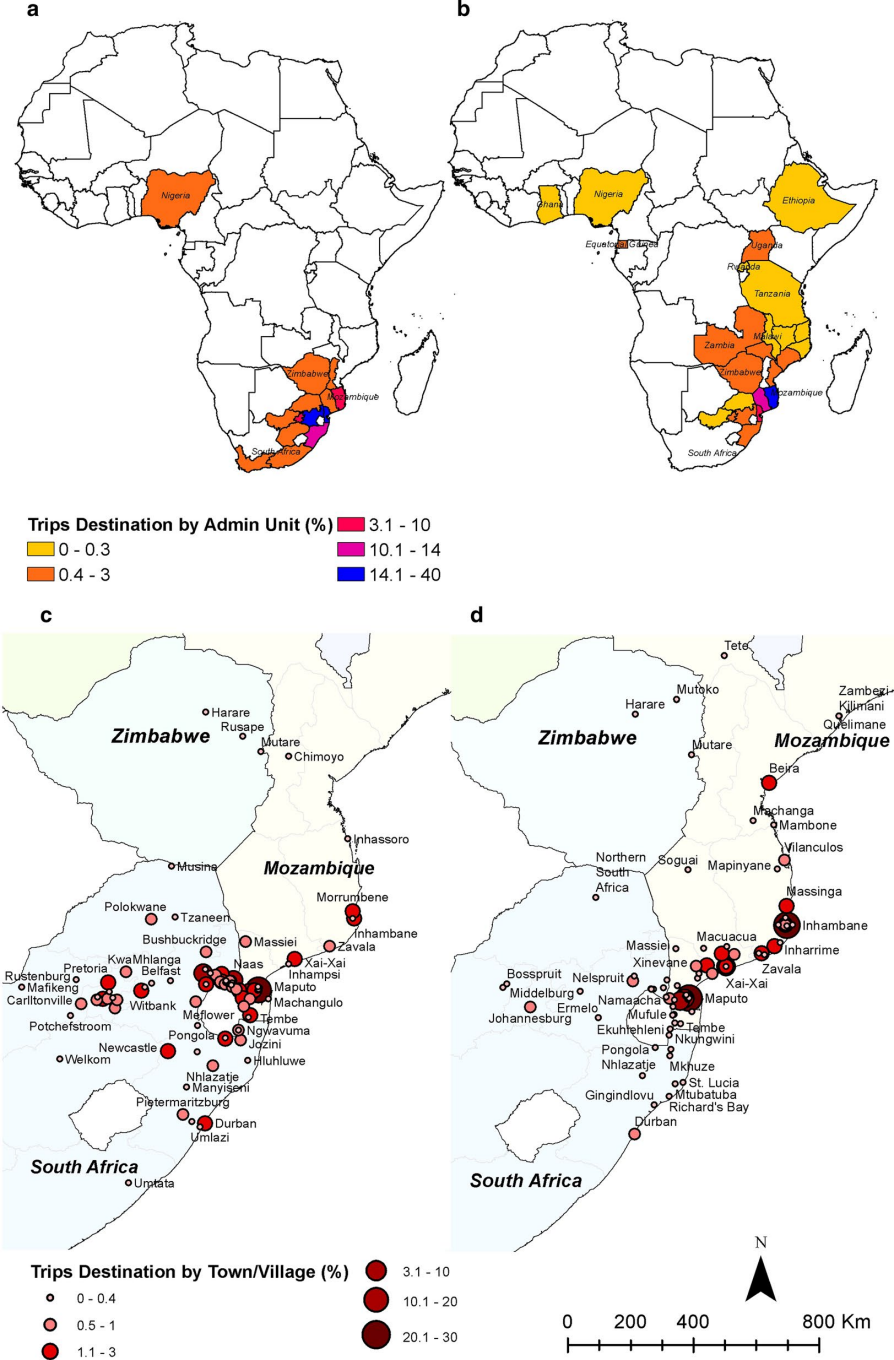
Travel patterns outside of Swaziland by negative cases (**a**) and (**c**) and positive cases (index and secondary) (**b**) and (**d**). Percentage of trips by administrative level 1 for South Africa and Mozambique are presented, and administrative level 0 for the rest of the countries in (**a**) and (**b**). Close-up of travel destinations nearest to Swaziland are shown by towns/cities in (**c**) and (**d**)

The top three most popular destinations for travel by those people tested in RACD and found to be negative, were Maputo City in Mozambique, with 86 (28%) trips, followed by Mpumalanga in South Africa with 76 (25%) trips, and Maputo province in Mozambique with 44 (14%) trips (Fig. 5a). The top three most popular destinations for those with confirmed malaria were all in Mozambique, in the regions of Maputo City with 315 (33%) trips, followed by Inhambane with 281 (30%) trips and Gaza with 123 (13%) trips (Fig. 5b). In general, there were substantial differences between the travel patterns of positive and negative cases. The travel histories of those with confirmed malaria included nine more countries and six more regions within Mozambique than the negative cases. The travel histories of the negative cases included three more regions within South Africa than the positive cases. Overall, 92% of the trips made by positive cases were made to Mozambique and 5% were to South Africa. In contrast, only 50% of the trips made by negative cases were to Mozambique and 48% were to South Africa.

**Fig. 6:**
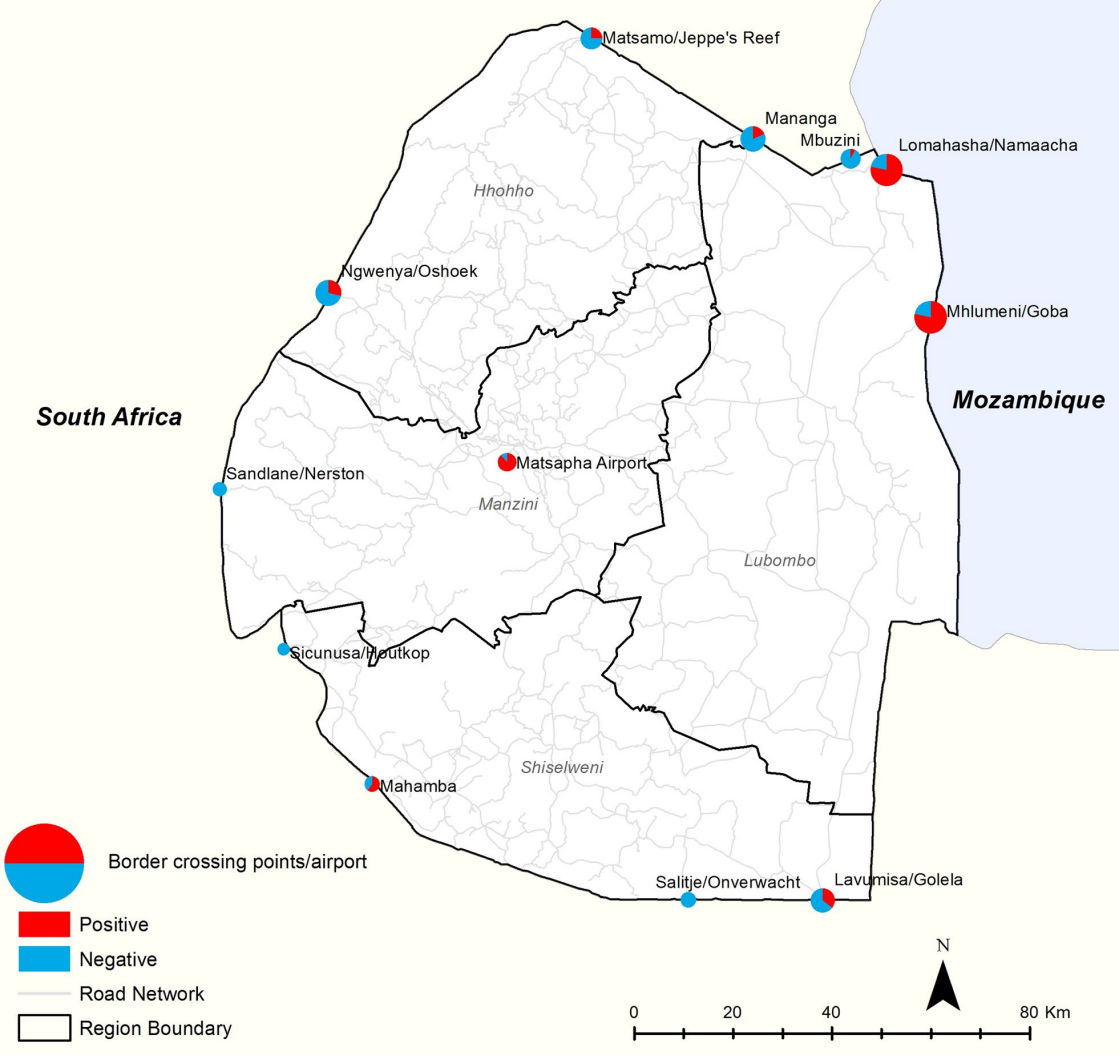
Border crossings with the percentage of investigated cases (index and secondary) that travelled abroad, scaled by the log of the total. Positive cases (n = 573) and negative cases (n = 306) are mapped separately. Informal crossings, other crossings and not reported crossings are not shown on the map

There were 25 unique border crossing points reported in the case investigations and, although for a large proportion of positive cases (39.4%) the crossing point was not recorded, the most popular crossings were two points bordering with Mozambique: Mhlumeni/Goba (27% of positive cases and 23% negative) and Lomahasha/Namaacha (26% positive and 23% negative); followed by three points bordering with South Africa: Ngwenya/Oshoek (2% positive and 12% negative), Mananga (0.7% positive and 10% negative), Lavumisa/Golela (1% positive and 7% negative); and informal crossings (1% positive and 7% negative), used by people travelling to Mozambique, South Africa and Nigeria (Fig. 6). The reported means of transport for those travelling internationally that were confirmed positive for malaria were kombi (van) (27%), large bus (15%), personal car (16%), walking (1.3%), ride share (1.1%), airplane (1%), truck (0.1%) and the remainder (39.4%) of the positive cases did not report a means of transport. Negative cases showed a similar pattern with kombi (van) (57.2%), large bus (19.6%), personal car (16.3%), walking (5.6%), airplane (0.7%), ride share (0.3%), bicycle (0.3%) and truck (0.1%).

### Malaria infection risk factors for international travel

The risk factors identified from univariate logistic regression of individuals (Table 1) and individual trips made outside the country (Table 2) indicate that there were strong differences in age and gender between those who were malaria-positive and -negative. Males had an increased risk with an OR of 3.65 CI 2.77–4.78 (p < 0.0001) when compared to females. Age classes of 1–4.9 and 24–44.9 years had an increased risk of being malaria positive with OR of 18.52 CI 5.28–76.75 (p < 0.001) and 8.31 CI 2.65–31.16 (p = 0.001) respectively, when compared to the <1-year old age class. The occupational groups that were associated with higher risks of being malaria positive, when compared to those unemployed, were: manufacturing (OR 7 CI 2.37–29.95, p = 0.002), office or clerical work (OR 4.38 CI 1.76–13.26, p = 0.003) and manual labour (OR 3.39 CI 2.07–5.74, p < 0.0001). From all the nationalities that were reported, Mozambicans showed a considerably increased risk of being malaria positive (OR 9.30 CI 6.16–14.64, p < 0.0001), when compared to Swazi nationals. The main risk factors that remained highly relevant in the multivariate analysis was being male, Mozambican and in the age class 1–4.9 and to a lesser extent being in the age classes of 5–14.9 and 25–44.9 (Table 1).

**Table 1:** Characteristics of all individuals that travelled outside Swaziland (positive and negative cases), with a percentage (%) of the total group size (n) and 95% confidence intervals (95% CI)

Variable	Characteristics				Risk factors				
	Positive cases		Negative cases		*p* (χ^2^)	OR (95% CI)	Pr(>|z|)	Adjusted OR (95% CI)	Pr(>|z|)
	n	% (95% CI)	n	% (95% CI)					
Total	902		297						
*Age class*					<0.0001				
<1	4	0.44 (0.01–0.88)	9	3.03 (1.08–4.98)		1		1	
1–4.9	107	11.86 (9.75–13.97)	13	4.38 (2.05–6.7)		18.52 (5.28–76.75)	<0.0001	24.74 (6.06–117.47)	<0.0001
5–14.9	92	10.2 (8.22–12.17)	44	14.81 (10.78–18.86)		4.7 (1.45–18.14)	0.0137	8.91 (2.15–42.79)	0.0037
15–24.9	100	11.09 (9.04–13.14)	68	22.9 (18.18–27.68)		3.31 (1.03–12.61)	0.0541	5.79 (1.5–26.11)	0.0144
25–44.9	384	42.57 (39.35–45.8)	104	35.02 (29.59–40.44)		8.31 (2.65–31.16)	0.0006	8.21 (2.22–35.61)	0.0025
45–64.9	98	10.86 (8.83–12.9)	45	15.15 (11.07–19.23)		4.90 (1.51–18.87)	0.0113	5.79 (1.47–26.43)	0.0155
65–99	12	1.33 (0.58–2.08)	14	4.71 (2.3–7.12)		1.93 (0.49–8.62)	0.3605	2.83 (0.56–15.58)	0.2136
Didn’t respond*	105	11.64 (9.55–13.73)	0						
*Occupation*					<0.0001				
Unemployed	144	15.96 (13.57–18.35)	126	42.42 (36.8–48.05)		1		1	
Agriculture	32	3.55 (2.34–4.75)	20	6.73 (3.88–9.58)		1.4 (0.77–2.6)	0.2778	0.75 (0.37–1.54)	0.4272
Manufacturing	24	2.66 (1.61–3.71)	3	1.01 (−0.13–2.15)		7 (2.37–29.95)	0.0018	3.06 (0.92–14.08)	0.0978
Office or clerical work	25	2.77 (1.7–3.84)	5	1.68 (0.22–3.16)		4.38 (1.76–13.26)	0.0035	1.96 (0.69–6.5)	0.2325
Other	108	11.97 (9.85–14.09)	37	12.46 (8.7–16.21)		2.55 (1.65–4.01)	<0.0001	1.14 (0.65–2.01)	0.6402
Other manual labour	93	10.31 (8.33–12.29)	24	8.09 (4.98–11.18)		3.39 (2.07–5.74)	<0.0001	1.09 (0.6–2.01)	0.7702
Small-market sales or trade	49	5.43 (3.95–6.91)	16	5.39 (2.82–7.96)		2.68 (1.48–5.08)	0.0016	1.71 (0.85–3.53)	0.1371
Student	74	8.2 (6.41–9.99)	66	22.22 (17.49–26.95)		0.98 (0.65–1.48)	0.9270	0.6 (0.31–1.17)	0.1382
Army	0		0						
Didn’t respond*	353	39.14 (35.95–42.32)	0						
*Gender*					<0.0001				
Female	262	29.05 (26.08–32.01)	178	59.93 (54.36–65.51)		1		1	
Male	636	70.51 (67.53–73.49)	119	40.07 (34.49–45.64)		3.65 (2.77–4.78)	<0.0001	4.67 (3.24–6.8)	<0.0001
Didn’t respond*	4	0.44 (0.01–0.88)	0						
*Nationality*					<0.0001				
Swazi	465	51.55 (48.29–54.81)	267	89.9 (85.76–92.97)		1		1	
Mozambican	405	44.9 (41.65–48.15)	25	8.42 (5.63–12.32)		9.30 (6.16–14.64)	<0.0001	4.88 (3.08–8)	<0.0001
South African	7	0.78 (0.2–1.35)	2	0.67 (0.12–2.68)		2.01 (0.48–13.56)	0.386	1.73 (0.33–13.18)	0.5430
Portuguese	1	0.11 (−0.11–0.33)	1	0.34 (0.02–2.16)		0.57 (0.02–14.55)	0.695	–	0.9948
Chinese	1	0.11 (−0.11–0.33)	0						
American	3	0.33 (−0.04–0.71)	0						
Malawian	4	0.44 (0.01–0.88)	0						
Zambian	1	0.11 (−0.11–0.33)	0						
Zimbabwean	5	0.55 (0.07–1.04)	2	0.67 (0.12–2.68)		1.49 (0.20–17.10)	0.667	1.81 (0.2–17.1)	0.5835
Indian	1	0.11 (−0.11–0.33)	0						
Rwandan	3	0.33 (−0.04–0.71)	0						
Congolese	1	0.11 (−0.11–0.33)	0						
Nigerian	1	0.11 (−0.11–0.33)	0						
Pakistan	1	0.11 (−0.11–0.33)	0						
Other	3	0.33 (−0.04–0.71)	0						

Risk factors are presented as odd ratios (OR) with 95% confidence intervals (95% CI) for the univariate model and adjusted OR when all variables were use in a multivariate model. *p* values from Wald test (Pr(>|z|)) for individual variables and *p* from X^2^ of the model tested against the null hypothesis. Those variables with (*) were not included in the models

**Table 2:** Characteristics of the trips made outside of Swaziland (positive and negative cases), with a percentage (%) of the total group size (n) and 95% confidence intervals (95% CI)

Variable	Characteristics				Risk factors		
	Positive cases		Negative cases		*p* (χ^2^)	OR (95% CI)	Pr(>|z|)
	n	% (95% CI)	n	% (95% CI)			
Total	947		306				
*Reason of travel*					0.003		
Lives/staying there	51	5.39 (3.94–6.82)	37	12.09 (8.44–15.74)		1	
Business	51	5.39 (3.97–6.82)	30	9.8 (6.47–13.14)		1.23 (0.67–2.3)	0.5063
Holiday	19	2.01 (1.11–2.9)	11	3.59 (1.51–5.68)		1.25 (0.54–3.02)	0.6049
Other	6	0.63 (0.13–1.14)	15	4.9 (2.48–7.32)		0.29 (0.1–0.79)	0.0194
Visiting friends/relatives	448	47.31 (44.13–50.49)	213	69.61 (64.45–74.76)		1.53 (0.96–2.4)	0.068
Didn’t respond*	372	39.28 (36.7–42.39)	0				
*Travel protection*					0.902		
Any protection	173	18.27 (15.81–20.73)	55	17.97 (13.67–22.28)		1	
No protection	773	81.63 (79.16–84.09)	251	82.03 (77.72–86.33)		0.98 (0.7–1.36)	0.902
Didn’t respond*	1	0.11 (−0.1–0.31)	0				
*Travel protection*					0.045		
Bed net	83	8.76 (6.96–10.57)	38	12.42 (8.72–16.11)		1	
None	773	81.63 (79.16–84.09)	251	82.03 (77.72–86.33)		1.41 (0.93–2.11)	0.10005
Repellent coil	28	2.96 (1.88–4.04)	9	2.94 (1.05–4.83)		1.42 (0.63–3.46)	0.41109
Chemoprophylaxis (chemop)	53	5.6 (4.14–7.06)	8	2.61 (0.83–4.4)		3.03 (1.37–7.45)	0.00934
Bed net + repellent coil	4	0.42 (0.01–0.84)	0				
Bed net + chemop	4	0.42 (0.01–0.84)	0				
Chemop + repellent coil	1	0.11 (−0.1–0.31)	0				
Didn’t respond*	1	0.11 (−0.1–0.31)	0				
*Bed net at home*					<0.0001		
Bed net	134	14.15 (11.93–16.37)	112	36.6 (31.2–42)		1	
None	813	85.85 (83.63–88.07)	194	63.4 (58–68.9)		3.5 (2.61–4.71)	<0.0001
*Admin unit/Country*					<0.0001		
KwaZulu-Natal (South Africa)	16	1.69 (0.87–2.51)	37	12.09 (8.44–15.74)		1	
Eastern Cape (South Africa)	0		1	0.33 (−0.31–0.97)			
Free State (South Africa)	0		1	0.33 (−0.31–0.97)			
Gauteng (South Africa)	4	0.42 (0.01–0.84)	22	7.19 (4.3–10.08)		0.42 (0.11–1.32)	0.162596
Limpopo (South Africa)	2	0.21 (−0.08–0.5)	7	2.29 (0.61–3.96)		0.66 (0.09–3.1)	0.628196
Mpumalanga (South Africa)	19	2.01 (1.11–2.9)	76	24.84 (20–29.68)		0.58 (0.27–1.26)	0.164403
North West (South Africa)	2	0.21 (−0.08–0.5)	3	0.98 (−0.12–2.08)		1.54 (0.19–10.18)	0.652284
Western Cape (South Africa)	0		1	0.33 (−0.31–0.97)			
Gaza (Mozambique)	123	12.99 (10.85–15.13)	5	1.63 (0.21–3.05)		56.89 (21.13–185.28)	<0.0001
Inhambane (Mozambique)	281	29.67 (26.76–32.58)	17	5.56 (2.99–8.12)		38.22 (18.26–84.63)	<0.0001
Manica (Mozambique)	0		1	0.33 (−0.31–0.97)			
Maputo (Mozambique)	71	7.5 (5.82–9.17)	45	14.71 (10.74–18.67)		3.65 (1.85–7.47)	0.000263
Maputo City (Mozambique)	315	33.26 (30.26–36.26)	86	28.1 (23.07–33.14)		8.47 (4.57–16.34)	<0.0001
Nampula (Mozambique)	2	0.21 (−0.08–0.5)	0				
Nassa (Mozambique)	1	0.11 (−0.1–0.31)	0				
Sofala (Mozambique)	14	1.48 (0.71–2.25)	0				
Tete (Mozambique)	3	0.32 (−0.04–0.67)	0				
Zambezia (Mozambique)	4	0.42 (0.01–0.84)	0				
Cabo Delgado (Mozambique)	1	0.11 (−0.1–0.31)	0				
Equatorial Guinea	4	0.42 (0.01–0.84)	0				
Ethiopia	1	0.11 (−0.1–0.31)	0				
Ghana	1	0.11 (−0.1–0.31)	0				
Nigeria	1	0.11 (−0.1–0.31)	1	0.33 (−0.31–0.97)		2.31 (0.09–60.96)	0.561948
Malawi	2	0.21 (−0.08–0.5)	0				
Rwanda	1	0.11 (−0.1–0.31)	0				
Tanzania	1	0.11 (−0.1–0.31)	0				
Zambia	4	0.42 (0.01–0.84)	0				
Zimbabwe	3	0.32 (−0.04–0.67)	3	0.98 (−0.12–2.08)		2.31 (0.39–13.71)	0.335023
Uganda	3	0.32 (−0.04–0.67)	0				
Didn’t respond*	68	7.18 (5.54–8.82)	0				
*Border crossing*					<0.0001		
Mananga	7	0.74 (0.32–1.59)	31	10.13 (7.09–14.21)		1	
Mhlumeni/Goba	255	26.93 (24.15–29.9)	70	22.88 (18.37–28.07)		8.99 (4.75–17.77)	<0.0001
Lomahasha/Namaacha	250	26.4 (23.64–29.35)	70	22.88 (18.37–28.07)		8.81 (4.66–17.43)	<0.0001
Ngwenya/Oshoek	15	1.58 (0.92–2.66)	37	12.09 (8.76–16.4)		0.56 (0.19–1.5)	0.25901
Lavumisa/Golela	12	1.27 (0.69–2.27)	22	7.19 (4.66–10.84)		1.35 (0.53–3.4)	0.52929
Informal crossings	9	0.95 (0.46–1.86)	21	6.86 (4.4–10.45)		1.06 (0.39–2.81)	0.91193
Matsamo/Jeppe’s Reef	5	0.53 (0.19–1.3)	15	4.9 (2.87–8.13)		0.82 (0.23–2.56)	0.74437
Mbuzini	1	0.11 (0.01–0.68)	11	3.59 (1.9–6.53)		0.22 (0.01–1.31)	0.16956
Matsapha Airport	8	0.84 (0.39–1.73)	1	0.33 (0.02–2.09)		19.73 (3.23–382.87)	0.0069
Salitje/Onverwacht	0		5	1.63 (0.6–3.99)			
Mahamba	3	0.32 (0.08–1)	2	0.65 (0.11–2.6)		3.7 (0.56–30.31)	0.17419
Sandlane/Nerston	0		4	1.31 (0.42–3.54)			
Sicunusa/Houtkop	0		3	0.98 (0.25–3.08)			
Other (total)^+^	8	0.84 (0.39–1.73)	14	4.58 (2.61–7.73)		1.41 (0.48–4.03)	0.52395
Other	3	0.32 (0.08–1)	3	0.98 (0.25–3.08)			
Sicancweni (other)	2	0.21 (0.04–0.85)	1	0.33 (0.02–2.09)			
Nsalitje (other)	0		2	0.65 (0.11–2.6)			
Phocweni (other)	2	0.21 (0.04–0.85)	0				
Tsambokhulu (other)	0		2	0.65 (0.11–2.6)			
Eticancweni (other)	0		1	0.33 (0.02–2.09)			
Insubane (other)	0		1	0.33 (0.02–2.09)			
Lubombo (other)	0		1	0.33 (0.02–2.09)			
Mahlahlane (other)	0		1	0.33 (0.02–2.09)			
Malabo international airport (other)	1	0.11 (0.01–0.68)	0				
Sicanco (other)	0		1	0.33 (0.02–2.09)			
Sitsasaweni (other)	0		1	0.33 (0.02–2.09)			
Didn’t respond*	374	39.49 (36.38–42.7)	0				
*Means of transport*					<0.0001		
Kombi (van)	256	27.03 (24.25–30)	175	57.19 (51.43–62.77)		1	
Large bus	139	14.68 (12.52–17.13)	60	19.61 (15.4–24.6)		1.58 (1.11–2.28)	0.0120
Airplane	9	0.95 (0.46–1.86)	2	0.65 (0.11–2.6)		3.08 (0.78–20.34)	0.1538
Bicycle	0		1	0.33 (0.02–2.09)			
Personal car	147	15.52 (13.31–18.02)	50	16.34 (12.47–21.07)		2.01 (1.39–2.94)	0.0003
Ride share	10	1.06 (0.54–2)	1	0.33 (0.02–2.09)		6.84 (1.29–126.02)	0.0680
Truck	1	0.11 (0.01–0.68)	0				
Walked	12	1.27 (0.69–2.27)	17	5.56 (3.37–8.91)		0.48 (0.22–1.03)	0.0614
Didn’t respond*	373	39.39 (36.27–42.59)	0				

Risk factors are presented as odd ratios (OR) with 95% confidence intervals (95% CI) for the univariate model. *p* values from Wald test (Pr(>|z|)) for individual variables and *p* from X^2^ of the model tested against the null hypothesis. Those variables with (*) were not included in the model. Note: some individuals carried out more than one trip. Variables with the (^+^) indicate the total of other border crossing points not geolocated, and identified as ‘other’ in brackets

Travelling to destinations, such as the Gaza region and Inhambane in Mozambique, meant an increased risk of infection, with ORs of 56.89 CI 21.13–185.28 (p < 0.0001) and 38.22 CI 18.26–84.63 (p < 0.0001), respectively, when compared against a reference travel destination of KwaZulu-Natal in South Africa. *Plasmodium falciparum* prevalences at the destination of travel estimated using malaria risk maps [5] showed generally higher prevalences at the locations visited by positive cases, compared to those testing negative (Table 3).

The use of a bed net as travel protection showed no substantial difference in the risk of infection compared to those who did not use one, although 81.6% of the cases that tested positive for malaria did not use travel protection. The use of chemoprophylaxis actually increased the risk of infection (OR 3.03 CI 1.37–7.45, p = 0.009) when compared to using a bed net and also when compared to not using any protective measures while travelling (OR 2.15 CI 1.07–4.95. p = 0.047). Not having a bed net at home increased the risk of infection (OR 3.5 CI 2.61–4.71, p < 0.0001) when compared to those who owned a bed net. Moreover, when comparing those who did not travel and had a bed net at home with those who travelled and did not have a bed net, the latter increased the risk of infection considerably (OR 62.38 CI 49.77–78.69, p < 0.0001) (Additional file 1: Table S2).

Border crossing points where individuals had a higher likelihood of returning positive for malaria, when compared to Mananga border crossing post between Swaziland and South Africa, were Mhlumeni/Goba (OR 8.99 CI 4.75–17.77, p < 0.0001) and Lomahasha/Namaacha (OR 8.81 CI 4.66–17.43, p < 0.0001), which are both border posts with Mozambique (Fig. 6). Departing via Matsapha airport was also a substantial risk factor, but with a large confidence interval (OR 19.73 CI 3.23–382.87, p = 0.006). The only means of travel that suggested to increase the risk of malaria was personal car (OR 2.019 CI 1.39–2.94, p = 0.003), when compared to using a Kombi (van).

### Travel within Swaziland

Some 888 people travelled within Swaziland (malaria prevalence of 20%), making 927 trips in total. For 813 (88%) of these trips the length of travel was reported, 9% spent a night away, 59% spent between two and ten nights away on their travels, 15% spent between 11 and 30 nights away and 9% between 31 and 100 nights, with 8% spending over 100 nights away. The most common regions of residence among those travellers who tested positive were Lubombo (49%) and Manzini (22%).

**Fig. 7:**
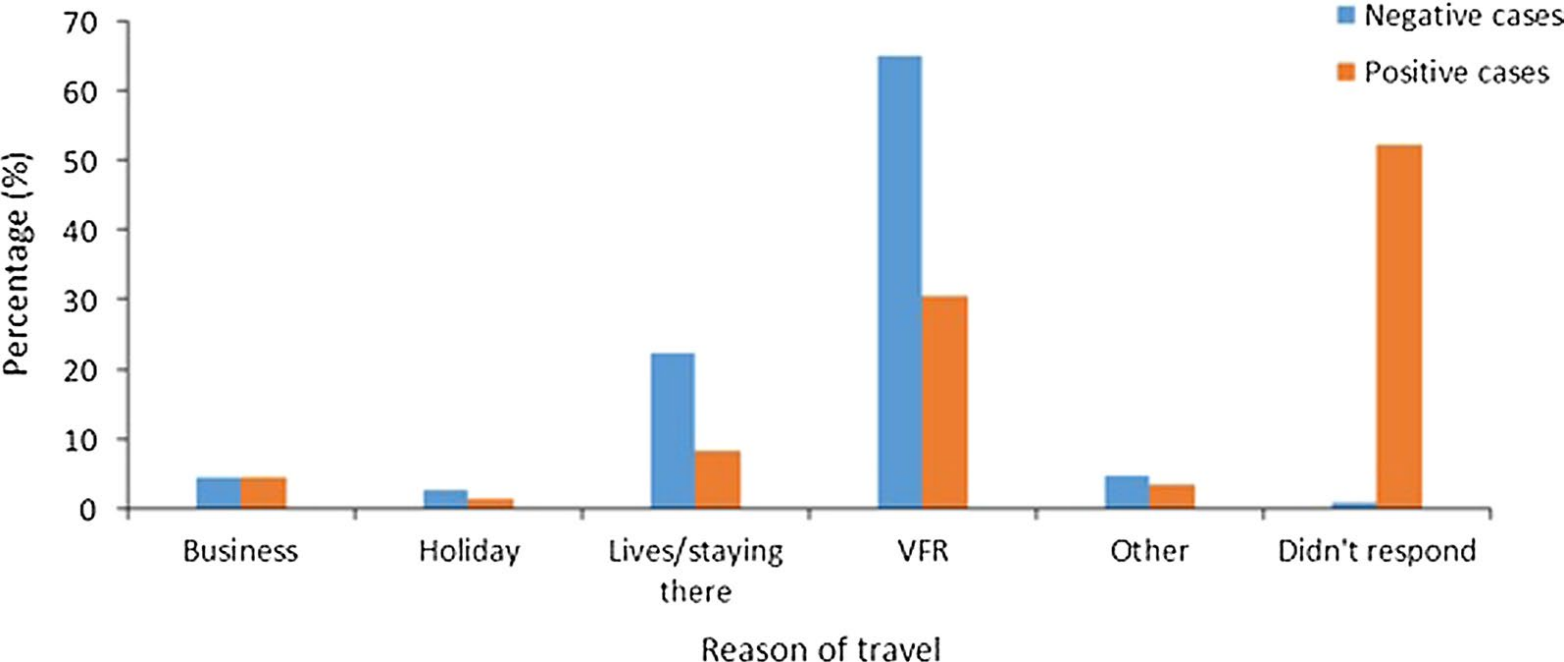
Reason for travel within Swaziland reported by those cases (index and secondary) that reported travel. Results are divided by negative (n = 720) and positive (n = 207) cases. *VFR* visiting friends and relatives. Further information of the classifications can be found in Additional file 1: Table S4

Overall the most common reason of travel was recorded as visiting friends/relatives (VFR) with 57% (Fig. 7). A large percentage of positive cases (52%) did not report the reason for travel; 19% lived or were staying in their destination. All the travel destinations were within 60 km of urban areas and <5% were within an urban area for both positive (2%) and negative cases (4%).

**Fig. 8:**
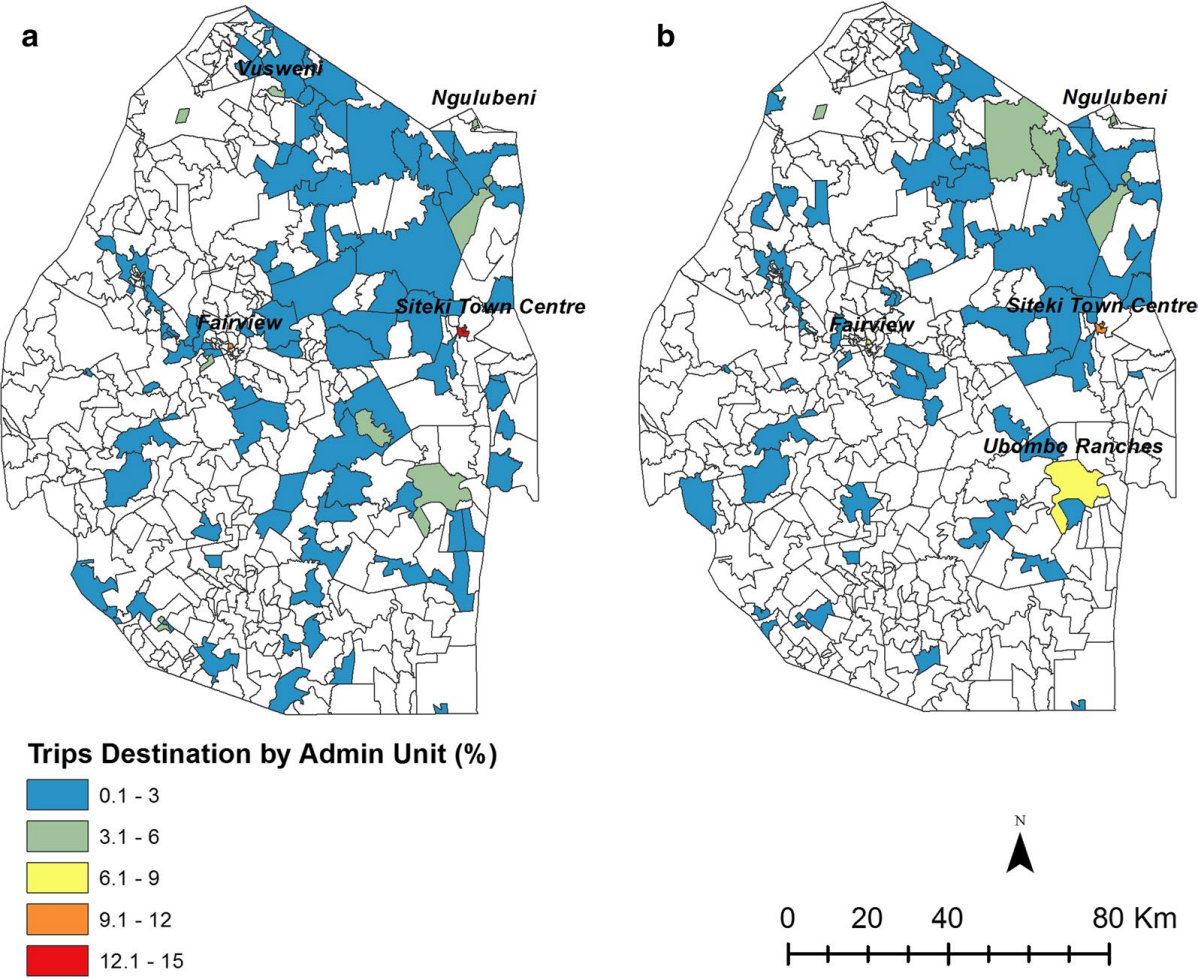
Destinations of domestic trips made by **a** negative and **b** positive cases from January 2010 to June 2014 mapped as percentage of total trips for negative (n = 714) and positive (n = 206) confirmed cases. The named locations are the most popular destinations

The destinations of domestic travel are shown in Fig. 8 for the positive and negative cases. Few differences were seen between the travel destinations of the positive and negative cases, with Siteki Town Centre in Lugongolweni (12%), Fairview in Manzini (9%), and Ngulubeni in Lomahasha (5%) being the most visited by those confirmed negative through RACD, and Siteki Town Centre in Lugongolweni (11%), Fairview in Manzini (7%), Ubombo Ranches in Nkilongo (7%) being the most visited by those with positive malaria confirmation.

**Table 3:** *Plasmodium falciparum* prevalence at destination of travel averaged by country, for all the trips made outside Swaziland by positive and negative cases

Country	Positive cases		Negative cases		Total	
	Mean *Pf* prevalence	Trips	Mean *Pf* prevalence	Trips	Total *Pf* prevalence	Total trips
Equatorial Guinea	0.4490	4			0.4490	4
Ethiopia	0.0000	1			0.0000	1
Ghana	0.2737	1			0.2737	1
Malawi	0.2822	4			0.2822	4
Mozambique	0.2302	873	0.1579	154	0.2187	1027
Nigeria	0.4901	1	0.2483	1	0.3692	2
Rwanda	0.0199	1			0.0199	1
South Africa	0.0522	46	0.0441	148	0.0460	194
Tanzania	0.1531	2			0.1531	2
Uganda	0.3196	3			0.3196	3
Unknown		1				1
Zambia	0.1028	6			0.1028	6
Zimbabwe	0.0516	4	0.0400	3	0.0458	7
Total	0.2213	947	0.1020	306	0.1905	1253

### Malaria infection risk factors for domestic travel

Of those 888 investigated individuals who reported travelling within Swaziland, 177 (20%) were malaria-positive cases and 112 (63%) of these were males, which was a higher proportion than the 45% out of those who were negative. From the univariate analysis, being male increased the risk of positive malaria confirmation (OR 2.10 CI 1.49–2.96, p < 0.0001) when compared to females. Being in an age class of 5–14.9 years increased the risk of infection (OR 9.29 CI 1.83–169.76, p = 0.03) when compared to <1-year olds. Being Mozambican also substantially increased the risk (OR 34.42 CI 6.25–640.85, p < 0.001), when compared to Swazi nationals. The occupational groups that increased the risk of infection, when compared to those cases that were unemployed, were: student (OR 2.41 CI 1.32–4.43, p = 0.004), office or clerical work (OR 5.57 CI 1.4–19.23, p = 0.008) and manual labour (OR 2.89 CI 1.06–7.12, p = 0.03). The main risk factors that remained highly relevant in the multivariate analysis was gender and to a lesser extent being an office or clerical work. Further information is shown in Table 4.

**Table 4:** Characteristics of individuals that reported a travel history that was within Swaziland by positive and negative cases, with a percentage (%) of the total group size (n) and 95% confidence intervals (95% CI)

Variable	Characteristics				Risk factors				
	Positive cases		Negative cases		*p*(χ^2^)	OR (95% CI)	Pr(>|z|)	Adjusted OR (95% CI)	Pr(>|z|)
	n	% (95% CI)	n	% (95% CI)					
Total	177		711						
*Age class*					0.0078				
<1	1	0.56 (−0.54–1.67)	29	4.08 (2.62–5.53)		1		1	
1–4.9	15	8.47 (4.37–12.58)	59	8.3 (6.27–10.33)		7.37 (1.39–136.55)	0.0587	5.34 (0.91–101.92)	0.1240
5–14.9	25	14.12 (8.99–19.26)	78	10.97 (8.67–13.27)		9.29 (1.83–169.76)	0.0324	4.57 (0.76–88.65)	0.1681
15–24.9	27	15.25 (9.96–20.55)	181	25.46 (22.26–28.66)		4.33 (0.87–78.63)	0.1578	3.18 (0.58–59.41)	0.2777
25–44.9	66	37.29 (30.16–44.41)	256	36.01 (32.48–39.53)		7.48 (1.55–134.43)	0.0498	2.96 (0.56–54.7)	0.3047
45–64.9	15	8.47 (4.37-12.58)	81	11.39 (9.06–13.73)		5.37 (1.02–99.25)	0.1109	2.25 (0.36–43.9)	0.4653
65–99	1	0.56 (−0.54–1.67)	19	2.67 (1.49–3.86)		1.52 (0.06–40.18)	0.7697	1.39 (0.05–38.01)	0.8252
Didn’t respond*	27	15.25 (9.96–20.55)	8	1.13 (0.35–1.9)					
*Occupation*					0.0038				
Unemployed	22	12.43 (7.57–17.29)	245	34.46 (30.97–37.95)		1		1	
Agriculture	10	5.65 (2.25–9.05)	85	11.95 (9.57–14.34)		1.31 (0.57–2.81)	0.5012	0.8 (0.33–1.85)	0.6123
Manufacturing	3	1.69 (−0.21–3.6)	7	0.98 (0.26–1.71)		4.77 (0.98–18.54)	0.0311	2.89 (0.57–11.7)	0.1549
Office or clerical work	4	2.26 (0.07–4.45)	8	1.13 (0.35–1.9)		5.57 (1.4–19.23)	0.0084	4.74 (1.13–17.54)	0.0231
Other	18	10.17 (5.72–14.62)	197	27.71 (24.42–31)		1.02 (0.53–1.95)	0.9582	0.6 (0.28–1.25)	0.1775
Other manual labour	7	3.95 (1.08–6.83)	27	3.8 (2.39–5.2)		2.89 (1.06–7.12)	0.0269	1.88 (0.65–5.04)	0.2236
Small-market sales or trade	0		12	1.69 (0.74–2.63)					
Student	27	15.25 (9.96–20.55)	125	17.58 (14.78–20.38)		2.41 (1.32–4.43)	0.0043	1.49 (0.65–3.36)	0.3373
Army	0		2	0.28 (−0.11–0.67)					
Didn’t respond*	86	48.59 (41.22–55.95)	3	0.42 (−0.05–0.9)					
*Gender*					<0.0001				
Female	64	36.16 (29.08–43.24)	388	54.57 (50.91–58.23)		1		1	
Male	112	63.28 (56.18–70.38)	323	45.43 (41.77–49.09)		2.10 (1.49–2.96)	<0.0001	2.77 (1.68–4.65)	0.0001
Didn’t respond*	1	0.56 (−0.54–1.67)	0						
*Nationality*					<0.0001				
Swazi	165	93.22 (89.52–96.92)	710	99.86 (99.58–100)		1		1	
Mozambican	8	4.52 (1.46–7.58)	1	0.14 (−0.13–0.42)		34.42 (6.25–640.85)	0.0009	–	0.9954
South African	1	0.56 (−0.54–1.67)		0					
Portuguese	1	0.56 (−0.54–1.67)		0					
Zimbabwean	2	1.13 (−0.43–2.69)		0					

Risk factors are presented as odd ratios (OR) with 95% confidence intervals (95% CI) for the univariate model and adjusted OR when all variables were use in a multivariate model. *p* values from Wald test (Pr(>|z|)) for individual variables and *p* from X^2^ of the model tested against the null hypothesis. Those variables with an (*) were not included in the models

The reason for travel that increased the risk ratio, compared to those who were living there or were staying there, was business (OR 2.66 CI 1.05–6.4, p = 0.03). Those trips in which travel protection was not used increased the risk of infection (OR 2.84 CI 1.48–6.17, p = 0.004), when compared to those using any type of protection. Not using a bed net at home increased the risk of infection (OR 1.88 CI 1.32–2.72, p < 0.001) (Table 5).

**Table 5:** Characteristics of the trips made within Swaziland, by positive and negative cases, with a percentage (%) of the total group size (n) and 95% confidence intervals (95% CI)

Variable	Characteristics				Risk factors		
	Positive cases		Negative cases		*p*(χ^2^)	OR (95% CI)	Pr(>|z|)
	n	% (95% CI)	n	% (95% CI)			
Total	207		720				
*Reason of travel*					0.2701		
Lives/staying there	17	8.21 (4.47–11.95)	161	22.36 (19.32–25.40)		1	
Business	9	4.35 (1.57–7.13)	32	4.44 (2.94–5.95)		2.66 (1.05–6.4)	0.0315
Holiday	3	1.45 (−0.018–3.08)	20	2.78 (1.58–3.98)		1.42 (0.31–4.71)	0.6001
Other	7	3.38 (0.92–5.84)	34	4.72 (3.17–6.27)		1.95 (0.71–4.91)	0.1704
Visiting friends/relatives	63	30.43 (24.17–36.70)	467	64.86 (61.37–68.35)		1.28 (0.74–2.31)	0.3952
Didn’t respond*	108	52.18 (45.37–58.99)	6	0.84 (0.17–1.50)			
*Travel protection (All)*					0.0011		
Any protection	9	4.35 (1.57–7.13)	82	11.39 (9.07–13.71)		1	
No protection	197	95.17 (92.25–98.09)	632	87.78 (85.39–90.17)		2.84 (1.48–6.17)	0.0038
Didn’t respond*	1	0.48 (−0.46–1.42)	6	0.83 (0.17–1.50)			
*Travel protection*					<0.0001		
Bed net	2	0.97 (−0.36–2.30)	80	11.11 (8.82–13.41)		1	
No protection	197	95.17 (92.25–98.09)	632	87.78 (85.39–90.17)		12.47 (3.88–76.21)	0.0005
Repellent coil	1	0.48 (−0.46–1.42)	2	0.28 (−0.12–0.66)		20 (0.75–337.99)	0.0347
Chemoprophylaxis (chemop)	6	2.9 (0.61–5.18)	0				
Didn’t respond*	1	0.48 (−0.46–1.42)	6	0.83 (0.17–1.50)			
*Bed net at home*					0.0004		
Bed net	47	22.71 (17–28.41)	256	35.56 (32.06–39.05)		1	
None	160	77.29 (71.59–83)	464	64.44 (60.95–67.94)		1.88 (1.32–2.72)	0.0006

Risk factors are presented as odd ratios (OR) with 95% confidence intervals (95% CI) for the univariate model. *p* values from Wald test (Pr(>|z|)) for individual variables and *p* from X^2^ of the model tested against the null hypothesis. Those variables with an (*) were not included in the model. Note: some individuals carried out more than one trip

## Discussion

Countries that are transitioning towards malaria elimination are improving their surveillance systems to capture remaining cases and gather more information about them. Robust analysis of these data is key to understanding the changing epidemiology of malaria and designing appropriate strategies to accelerate towards elimination. For countries that border neighbours with higher levels of transmission and have significant rates of cross-border travel, there is a need to understand rates of importation and risk factors for travellers to inform strategies. This research presents approaches to analysing the socio-demographic and travel profiles for each passively detected case recorded in Swaziland, and both the positive and negative cases identified during RACD. The results further the understanding of factors that contribute to the likelihood of acquiring malaria in Swaziland, and provide a detailed account of the most popular travel origin and destinations, highlighting the increasing trend in case importation as a result of travel to provide evidence for designing targeted strategies.

The results presented in this analysis highlight the burden that international travel to areas of higher transmission continues to have on the importation of malaria into Swaziland, being major challenge in sustaining malaria control and elimination [25]. The high number of imported and locally transmitted cases recorded consistently during the first few months of the year is likely caused by workers from Mozambique returning to Swaziland in January following the Christmas and New Year holidays [17]. Additionally, high importation rates have been attributed to sugar plantation workers whose travel patterns are well known between these two countries. Unsurprisingly, international travellers tend to spend longer away than domestic travellers, and given the higher risks of acquiring malaria in Mozambique, where the majority of travellers go, this length of stay increases the risks of acquiring and returning with parasites [26]. Differences were seen in travel patterns between genders, age groups and occupations. The reasons behind these are many and varied, but likely include adolescent age groups travelling to and from boarding schools and employed males travelling for work at plantations [16].

Additionally, this research examined the travel patterns of those who tested negative for malaria in RACD, enabling a comparison with the patterns presented by those who tested positive. The primary risk factor for malaria infection in Swaziland was shown to be travel (Additional file 1: Table S1), more specifically international travel to Mozambique by 25- to 44-year old males, who spent on average 28 nights away. Males that were malaria positive travelled to a total of 12 different countries, double the number visited by positive females; 67% of the reported international travel by positive males were carried out between December and March, which was higher than the 60% of international travel reported by females who tested positive during the same period; 96% of RDT-positive international travellers were either Swazi (52%) or Mozambican (45%) nationals, however Swazis were more likely to test negative.

Focused screening and treatment at borders has shown that malaria cases can be identified before reaching receptive areas [27], and this could be a viable option at those crossing points with the largest number of malaria positive cases such as those at the border with Mozambique at Mhlumeni and Lomahasha, targeting specific demographic groups such as males aged 25–44 that have travelled to areas of high malaria transmission and at specific times of year when the largest influx of people occurs. It is however important that robust public-acceptability and cost-effectiveness studies on such interventions be conducted first, as considering the use of RDTs for this purpose for example, may not be as efficient at identifying cases when the levels of parasites are low. Focussed screening could also be strengthened by network sampling, where tested individuals can help recruit others with similar travel characteristics that can potentially be at high risk of importing parasites [13, 28].

The current surveillance system of Swaziland’s NMCP, that incorporates RACD, shows the ability to detect cases that would otherwise be missed by passive surveillance [29]; however, additional efforts could be targeted to those high-risk groups, such as international travellers to high transmission areas, through education of the risks and provision of chemoprophylaxis, ensuring that that they are protected prior to travel, and adhering to the drug regimen properly. The availability of individual-level travel history data here allows for the identification of areas that have an elevated risk of malaria importation. Combining this information with high resolution predicted malaria incidence and health access maps [30] can provide guidance on targeting effective intervention packages by space, time and within populations.

There are limitations to this study, in particular the classifications of imported versus local cases, where a travel history prior to case confirmation does not necessarily mean that the case was imported. Additionally, assigning a likely location of case acquisition is uncertain when a patient travelled to multiple locations. The surveillance data and the changes within it during the time period covered also resulted in some uncertainties. The RACD was carried out in the latter half of the study period and was mainly focused on receptive areas, therefore there is a lack of information on negative cases and any potential secondary cases from other areas of the country. The length of time since recorded travel changed through time, as follows: January 2010–June 2010: travel within last 2 weeks; July 2010–June 2013: travel within last 4 weeks; and July 2013–June 2014: travel within last 8 weeks. Therefore, an increase in number of trips may have inflated the trend towards the latter part of the study. These changes occurred throughout the study in order to improve the quality of data. The use of RDTs may not have been the most sensitive method to identify malaria cases, as recent studies determined that RDTs have shown low sensitive owing to an unexpectedly high proportion of low-density infection among symptomatic subjects [31], but RDTs have some advantages over other methods, not only because they give a relatively rapid results, but because they require minimal training to use, there is little or no manipulation of the sample required and most RDTs do not require refrigeration, making it easier to use in areas where there is no power supply [32]. Interpretation of results should also consider classifications such as age classes, employment and reason of travel, as these were grouped here for comparative purposes. Furthermore, the index case classification was a subjective classification made by the NMCP’s Chief Surveillance Officer based on the likelihood of the origin of the case with reference to travel history. Throughout the study the number of unknown cases increased and these were mostly (72%) people that did not travel. The logistic regressions carried out in this analysis used specific reference groups to compare the likelihood of acquiring malaria based on known groups that were the least likely to test positive or had the majority of negative cases, so it is possible that different results could have been obtained if these reference groups varied, additionally, some variables were too small to develop meaningful multivariate models to describe risk factors. Future work will focus on a number of directions. The analyses will be updated as new surveillance data continue to be collected to provide contemporary evidence based upon which to guide programmatic decisions. The data will also provide a valuable input to modelling efforts to understand the effects of individual-level characteristics and spatial importation risk, e.g., where imported cases most often appear and their likelihood of prompting onward transmission to refine existing estimates and risk maps [18, 33]. The data could also prove useful in parameterizing models of human and parasite mobility that can be extended beyond Swaziland, as well as compared to other forms of mobility data, such as from mobile phones [7], census microdata [33, 34] or parasite genotyping [35] and, to strengthen assessments of the impact of climatic variations and incidence on malaria transmission [36] or genetic studies that further the understanding of transmission dynamic in low transmission settings [18].

The information provided in this analysis is useful to direct national and regional targeting of interventions, over space, time and by population groups. Results presented here highlight how Swaziland can continue to benefit from cross-border collaboration to manage importation of malaria at the regional level. Previous regional schemes, such as the Lubombo spatial development initiative (LSDI), made significant contributions to the reduction of malaria prevalence, with the introduction of effective treatment and vector control in the region [13]. The establishment of the elimination eight (E8) initiative, which aims to eliminate malaria from eight southern African countries, including Swaziland, South Africa and Mozambique by 2030 [2], will continue such collaborations as well as the revival of the LSDI as the Mozambique, South Africa and Swaziland (MOSASWA) Malaria cross-border initiative.

### Box 1 Summary of key findings

- This analysis highlights the impact that travel has on the incidence of malaria in Swaziland, in particular international travel to and from neighbouring countries with higher malaria prevalence.
- The main risk factors that remained highly relevant in a multivariate analysis for international travel were being male, Mozambican and in the age class 1–4 years old and to a lesser extent being in the age classes of 5–14 and 25–44 years.
- Not having travel protection measures as well as not having bed nets at home increase the risk of malaria infection.
- The following patterns were observed among those who tested malaria positive in Swaziland, as identified through passive and reactive case detection (RACD):
  - The main reason for domestic and international travel was to visit friends and relatives.
  - The top three most popular international destinations of travel were all in Mozambique, in the regions of Maputo City, followed by Inhambane and Gaza.
  - The most popular border crossings points where those bordering Mozambique: Mhlumeni/Goba and Lomahasha/Namaacha. With kombi (van), large bus and personal car, being the most popular choice of travel.
  - The most popular destinations of travel within the country were Siteki Town Centre in Lugongolweni and Fairview in Manzini.

## Additional file

**Additional file 1: Table S1.** Characteristics for all the individuals, positive and negative cases, with a percentage (%) of the total group size (n) and 95% confidence intervals (95% CI). Risk factors are presented as odd ratios (OR) with 95% confidence intervals (95% CI) for the univariate model and adjusted OR when all variables were use in a multivariate model. *p* values from Wald test (Pr(>|z|)) for individual variables and *p* from X^2^ of the model tested against the null hypothesis. Those variables with asterisk (*) were not included in the models. **Table S2.** Characteristics of all individual trips made by those who travelled within/outside of Swaziland and those who did not travel, by positive and negative cases, with a percentage (%) of the total group size (n) and 95% confidence intervals (95% CI). Risk factors are presented as odd ratios (OR) with 95% confidence intervals (95%CI) for the univariate model. *p* values from Wald test (Pr(>|z|)) for individual variables and *p* from X^2^ of the model tested against the null hypothesis. Those variables with asterisk (*) were not included in the model. Note: some individuals carried out more than one trip. **Table S3.** Characteristics for all the individuals that did not travel, positive and negative cases, with a percentage (%) of the total group size (n) and 95% confidence intervals (95% CI). Risk factors are presented as odd ratios (OR) with 95% confidence intervals (95% CI) for the univariate model and adjusted OR when all variables were use in a multivariate model. *p* values from Wald test (Pr(>|z|)) for individual variables and *p* from X^2^ of the model tested against the null hypothesis. Those variables with asterisk (*) were not included in the models. **Table S4.** Descriptions and classifications of the variables.

## Abbreviations

RDT: rapid diagnostic test
RACD: reactive case detection
NMCP: National Malaria Control Programme
MSDS: Swaziland Malaria Surveillance Database System
GADM: Global Administrative Areas Database
OR: odds ratio
CI: confidence intervals
LSDI: Lubombo spatial development initiative
E8: elimination eight
MOSASWA: Mozambique, South Africa and Swaziland
